# The Prediction in Computer Color Matching of Dentistry Based on GA+BP Neural Network

**DOI:** 10.1155/2015/816719

**Published:** 2015-03-22

**Authors:** Haisheng Li, Long Lai, Li Chen, Cheng Lu, Qiang Cai

**Affiliations:** ^1^School of Computer and Information Engineering, Beijing Technology and Business University, Beijing 100048, China; ^2^Peking University School and Hospital of Stomatology, Beijing 100081, China

## Abstract

Although the use of computer color matching can reduce the influence of subjective factors by technicians, matching the color of a natural tooth with a ceramic restoration is still one of the most challenging topics in esthetic prosthodontics. Back propagation neural network (BPNN) has already been introduced into the computer color matching in dentistry, but it has disadvantages such as unstable and low accuracy. In our study, we adopt genetic algorithm (GA) to optimize the initial weights and threshold values in BPNN for improving the matching precision. To our knowledge, we firstly combine the BPNN with GA in computer color matching in dentistry. Extensive experiments demonstrate that the proposed method improves the precision and prediction robustness of the color matching in restorative dentistry.

## 1. Introduction

**Figure 1:**
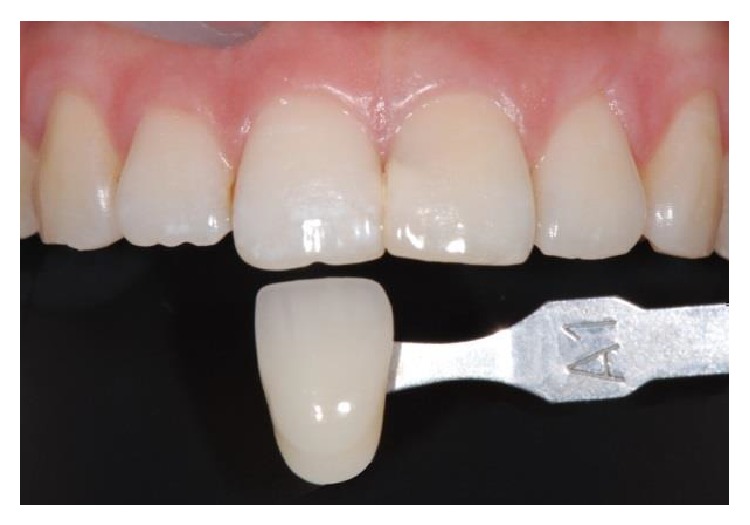
Color difference between target tooth and a shade guide tab.

With the rapid development of technology, various new materials are brought into dentistry. People no longer only pay attention to the functional recovery such as chewing and durability; instead they pay more attention to aesthetics [1]. Color is one of the key elements in determining the esthetics of ceramic restorations. Presently, the majority of shade selection is finished by visual assessment using shade guide tabs [2]. However, the color difference of target tooth and shade guide tab is not negligible, as shown in Figure 1, which leads shade selection to a formidable task. Intuitively, accurate shade selection may be the most important factor in esthetic restorative density. Visual selection is subjective and color distribution of shade guide tabs cannot cover those of natural teeth. Making accurate shade selection, along with proper materials and reasonable configuration, so that it can be consistent with the natural tooth color, is an urgent task that remains to be solved [3].

The computer color matching (CCM) technique provides the color matching of teeth restoration with a broad new method for research and application. Along with the Kubelka Munk theory put forward in 1931, computer color matching had been widely used in dyeing and printing industry. In a series of research from 1992 to 1994, Ishikawa-Nagai et al. realized computer color matching of opaque layer on the color of porcelain-fuse-to-metal restorations (PFM) using spectrophotometer [4–6]. Wang et al. conducted a feasibility study for CCM [7] and the results showed that the color repetition rate of front teeth restoration made by CCM outperforms the visual shade selection method [8].

It is worth mentioning that there is obvious chromatism between part of the porcelain pieces and natural dentition in the CCM based experiments by Ishikawa-Nagai et al. [9]. He analyzed the fact that the measurement of calculating some porcelain powders may result in deviation which affected the final precision. To solve the problem existing in Kubelka Munk theory based CCM, nonlinear methods, such as artificial neural network, are adopted in making porcelain restoration. In 2003, Wu et al. adopted BP algorithm in oil paint color design [10]. In 2008, Zhang et al. employed BP neural network with genetic algorithms in textile color matching and achieved good performance [11].

BP neural network is one of the most popular neural network methods presently [12]. However, the existing improved BP neural network has some drawbacks, such as low convergence rate [13, 14] and being difficult to devise suitable network structure [15]. In dentistry, for actual clinical application, high accuracy and good stability are the prerequisite for computer color matching. The success of computer color matching can greatly raise the work efficiency of dentists and technicians. In our study, we conduct many experiments to determine the structure of BP neural network. In addition, we introduce the genetic algorithm (GA) into the improved BP algorithm for assistance of computer color matching in dentistry [16–18]. Extensive experiments demonstrate that the accuracy of the GA+BP outperforms the state-of-the-art methods.

## 2. Algorithms

### 2.1. Back Propagation Neural Network (BPNN)

**Figure 2:**
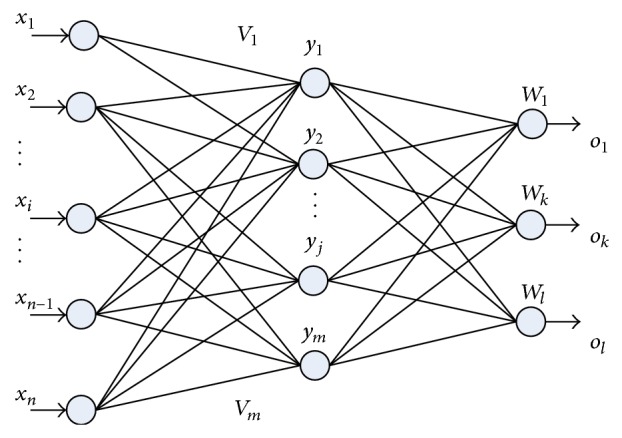
The BPNN structure with 3 layers. *x*_*i*_ is the input data set; *y*_*j*_ is hidden layer node; *o*_*k*_ is the actual output; *W* and *V* are the weights.

Artificial neural network (ANN) is accepted as a technology offering an alternative way to simulate complex and ill-defined problems. Back propagation neural network (BPNN) is a typical ANN that has been widely used in many medical fields such as medical image analysis, expert system for clinical diagnosis and treatment, medical signal analysis, and processing. It has successfully solved many complicated nonlinear problems. BPNN has hierarchical feed forward network architecture, and the outputs of each layer are sent directly to each neuron of the previous layer. BPNN can have many layers while all pattern recognition and classification tasks can be accomplished with a three-layer BPNN, as shown in Figure 2.

### 2.2. Disadvantage and Improvement of BPNN

According to Kolmogorov theorem and BP fix quantification, three-layer BP network with nonlinear excitation function can approach any nonlinear function at any precision. Multilayer perceptron is widely employed due to this remarkable advantage. However, the standard BP algorithm has some defects as follows.

1. In mathematics, it can be seen as a nonlinear gradient optimization problem. Therefore, it is easy to fall into local minima and cannot reach the global optimal solution.
2. Too much training makes convergent velocity slow.
3. It is difficult to determine the structure of hidden layer nodes due to lacking of theoretical guidance.
4. There exists tendency to forget old samples during training with new samples.

Aiming at these problems, three kinds of commonly used methods have been proposed.

*(1) Appending Momentum Item. *In order to improve the training velocity of the BPNN, a momentum item can be added in the weight adjustment formula. The weights adjustment vector expression with momentum item is shown in formula

(1) $$\begin{array}{c} \Delta W\left(t\right)=\eta \delta X+\alpha \Delta W\left(t-1\right). \end{array}$$

The formula shows that part of prior weight adjustment quantity will be added to current weight. The *α* is called momentum coefficient (normally *α* ∈ (0,1)). The momentum item reflects the prior adjustment experience. And it can reduce the vibrate trend when there is a sudden fluctuation of error curved surface. It can also improve the training velocity.

*(2) Adaptive Adjusting Learning Efficiency.* Learning efficiency is set to be constant in the standard BP algorithm. However, in practice, it is better to change learning efficiency according to the error.

An initial learning efficiency should be set. After a round of weight adjustment, if the total error increases, current adjustment is regarded as invalid, and adjust learning efficiency according to formula

(2) $$\begin{array}{c} \eta \left(t+1\right)=\beta \eta \left(t\right),\;\left(\beta <1\right). \end{array}$$

Whereas, if the total error descends, current adjustment is regarded as valid, meanwhile, adjust learning efficiency according to formula

(3) $$\begin{array}{c} \eta \left(t+1\right)=\theta \eta \left(t\right). \end{array}$$

*(3) Introducing Gradient Factor*. The reason why the weight adjustment is caught in the flat area is that the neurons' output is caught in the saturated zone of excitation function. In order to make the output away from the saturated zone, neuron net input should be compressed. Hence, the original excitation function can add a gradient factor *λ*, as shown in formula

(4) $$\begin{array}{c} o_{k}=\frac{1}{1+e^{-{\text{net}}_{k}/\lambda }}. \end{array}$$

It is considered to have entered the flat area when Δ*E* approximate to 0; nevertheless, *d*_*k*_ − *o*_*k*_ is still large. In this case, *λ* should be set to greater than 1, and after deviating from the flat area, *λ* should be set to 1 again.

### 2.3. Computer Color Matching of Restoration with GA+BP

The initial weights and threshold of traditional neural network are randomly generated. In addition, network connection weights and threshold of the whole distribution will influence the effect of data fitting. Improper initial parameters can lead to no convergence or fall into local extremum which will worsen the accuracy of the final prediction.

In clinical applications, it is needed to provide better service to patients with low error and high stability. Genetic algorithm (GA) is adopted to improve the accuracy of computer color matching of restoration. GA will optimize the initial weights and threshold values. It can effectively reduce the randomness of initial parameters. The local optimal defects of BP algorithm will be overcome due to more stable predictive effect by using GA and neural network.

**Figure 3:**
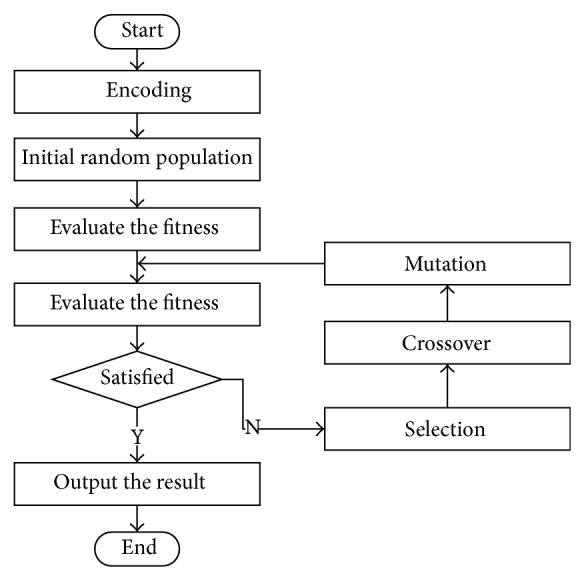
Flow chart of genetic algorithm.

Genetic algorithm is a simulated evolutionary process method. It follows the principle of evolution and takes the good individual evolution as the optimal solution. The flowchart of genetic algorithm is shown in Figure 3.

Each step of the genetic algorithm is explained as follows.

(1) Encoding, initial random fitness: using GA, each individual of the population needs to be described in a chromosome representation. Chromosome is composed of a series of real numbers. The encoded string consists of four segments, namely, the connection weights between hidden layer and input layer, the connection weights between output layer and hidden layer, hidden layer threshold, and output layer threshold.

(2) Fitnessfunction: GA uses fitness function to evaluate the viability of the chromosome. The fitness is corresponding with the error of BP neural network between the actual output and desired output. When the error is small, the fitness will be high.

(3) Selection: the extremely important step in GA is the selection. Selection is based on the fitness of each individual. In this paper, Roulette wheel selection is employed. A probability *p*_*i*_ will be evaluated for each individual *i*. The *p*_*i*_ is defined in formula

(5) $$\begin{array}{c} p_{i}=\frac{f_{i}}{\sum_{i=1}^{N}f_{i}}, \end{array}$$

where *f*_*i*_ is the fitness of individual *i* and *N* is the size of the population.

The probability of individual *i* to be selected is even greater when *p*_*i*_ is larger.

(4) Crossover and mutation: both the crossover and mutation can create new individuals by recombining or mutation. Crossover operation is to change corresponding segment of two individuals to get two new individuals. Mutation will return new individuals by altering the value of some elements of the chromosome.

(5) The process will be accomplished when we get appropriate fitness or evolution has completed the default maximum number of generations. The output of this process is the individual with best fitness and this individual consists of the weights and threshold. The weights and threshold will be used as the initial setup to train the BPNN.

## 3. Experiments

### 3.1. Train/Test Samples

**Figure 4:**
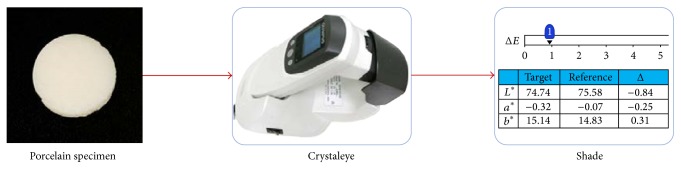
Measure of the shade of the specimen with crystaleye.

We mixed VITA VMK95 dentin porcelain powder according to different proportion. The powder will be molding in homemade stainless steel mold (the diameter is 15 mm; the thickness is 3 mm). Then, we put the porcelain powder into porcelain pieces in porcelain furnace and manufacture porcelain pieces of specimen. Finally, the color of porcelain restoration database is generated by measuring the shade of the specimen with crystaleye dental spectrophotometer [19], as shown in Figure 4.

Now a total of 119 sets of data by using the above-mentioned method have been obtained. The 75% of the data is used as training data set while the 25% of the data is used as test data set. The example of experimental data is shown in Table 1. *L* ^*^, *a* ^*^, and *b* ^*^ are converted from spectrum of visible light measured by crystaleye dental spectrophotometer colorimetric instrument. *A*1, *A*4, *B*4, *C*4, and *D*4 are different kinds of VITA VMK95 dentin porcelain powder. Different ratio of porcelain powder component can form corresponding color after burning. *L* ^*^, *a* ^*^, and *b* ^*^ are treated as input, while *A*1, *A*4, *B*4, *C*4, and *D*4 as the output. Each dimension of the input data needs to be normalized before training.

### 3.2. The Construction of BPNN Model

According to actual situation in the previous section, the number of nodes in input layer is 3 and it is 5 in output layer. As a result of the multihidden layers network structure is more complicated and the three layers of neural network can implement almost all pattern recognition and classification tasks; three-layer neural network is employed.

How to choose the number of hidden layer nodes has not been solved with a good analytic expression. The number of hidden layer nodes is often determined by the experience or testing.

Formula (6) is widely used for estimation of hidden layer nodes, and the final results will be determined through a set of experiments:

(6) $$\begin{array}{c} h=\sqrt{n+m}+a. \end{array}$$

In formula (6), *h* is the number of hidden layer nodes. *n* is input layer nodes. Output node number is *m*. And *a* is a constant integer (*a* ∈ [1,10]). Then we can get that the value of *h* is between 4 and 13.

**Figure 5:**
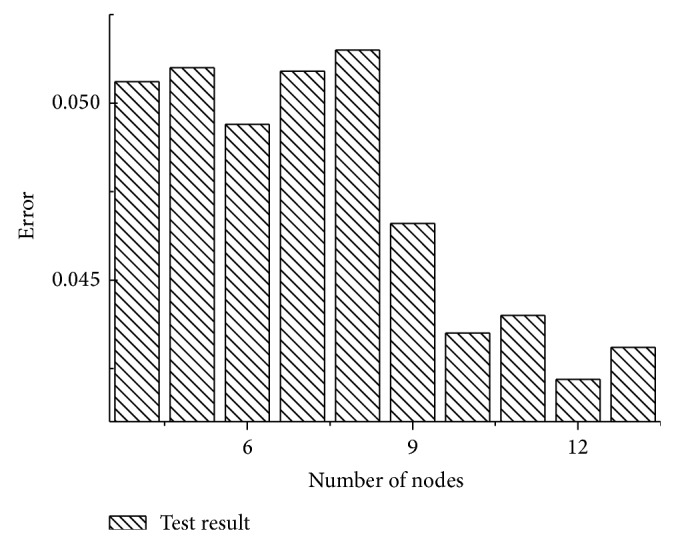
Comparisons of predictive ability of BPNN with different number of hidden layer nodes.

In order to get the specific number of hidden layer nodes, we introduced the ideas of trial and error and conducted a series of 10 trials. Each trial of test performed 20 times of prediction. The experimental data is training data set referred to in the previous section. Different trials have different hidden layer nodes while other parameters in different trials are consistent. The experiment results are shown in Figure 5.

As shown in Figure 5, it is obvious that the error is smallest when the number of hidden layer nodes is 12. Therefore, the number of hidden layer nodes is set to 12. Then, the construction of BPNN model is finished.

### 3.3. Application

#### 3.3.1. The Improved BPNN

After the network structure is identified, we conducted BPNN prediction experiments by using MATLAB neural network toolbox. The toolbox provides us with a variety of improved algorithms. Our statement of building the training model is as below:

 net = newff(inputn, outputn, hiddennum, {“tansig”, “tansig”}, “traingd”).

We can see from the above function that the two excitation functions are both tangent *S* transfer function. Our training function is “traingdx.” Namely, we adopt the improved BP algorithm with appending momentum item and adaptive adjusting learning efficiency. Examples of actual output and expected output of experiment are shown in Table 2.

We can use formula (7) to evaluate the error of each sample:

(7) $$\begin{array}{c} E^{p}=\sqrt{\overset{l}{\underset{k=1}{\sum }}{\left(d_{k}^{p}-o_{k}^{p}\right)}^{2}}. \end{array}$$

At last, the mean square error (MSE) is used to represent the total error of this structure. MSE is calculated by using formula

(8) $$\begin{array}{c} E_{\text{MSE}}=\sqrt{\frac{1}{P}\overset{p}{\underset{k=1}{\sum }}{\left(E^{p}\right)}^{2}}. \end{array}$$

We conducted a series of 10 tests. All tests have the same parameters and MSE of tests is shown in Table 3.

#### 3.3.2. GA+BP

The fitness function of GA algorithm is the BP algorithm provided by MATLAB neural network toolbox. We choose the Levenberg-Marquardt algorithm as the training function [20]. Levenberg-Marquardt algorithm for medium-sized BP neural network is the default training function of the toolbox, and it also has the fastest convergence speed.

The initialization parameter, namely, the threshold and weights, can be obtained after the GA process. Then, BPNN model with the initialization parameters is constructed. In this section, we chose the appending momentum item and introduced gradient factor to improve the BPNN. Similarly, we conducted a series of 10 trials. And we got the MSE of each group finally. The MSE of experiment in each trial is shown in Table 4.

### 3.4. Discussion

**Figure 6:**
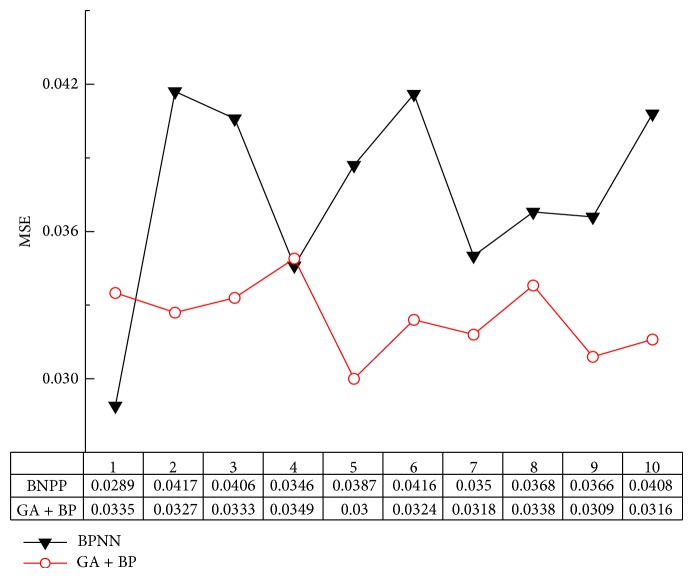
Comparisons of experimental results.

Comparisons between BPNN and GA+BP are shown in Figure 6. It is clear that the MSE of proposed GA+BP is generally smaller than that of BPNN. Furthermore, the prediction ability of GA+BP is more stable.

## 4. Conclusion

A more perfect forecasting model for dental porcelain computer color matching called GA+BP is proposed. Based on the research and comprehensive discussion about the traditional BPNN, the initial weights and threshold are optimized by GA firstly. Experiments show that it enhances the convergence performance and stability of the BPNN by determining the appropriate initial parameters instead of random selection of initial parameters. It makes the color matching of restoration more objective and accurate.

The GA+BP can help reach the prediction goal of CCM in actual research. Therefore it has high practical application value and plays a guidance role in CCM. With the development of computer science comprehensively introduced into the medical field, stomatological hospital will have more ability to provide better services for patients in the future.

## Figures and Tables

**Table 1:** Example of experimental data.

*L* ^*^	*a* ^*^	*b* ^*^	*A*1	*A*4	*B*4	*C*4	*D*4

66.89667	−0.01	14.92667	0.32	0	0	0.08	0
72.46333	−1.33333	14.58667	0.32	0	0	0	0.08
65.74667	1.33	19.24333	0.16	0	0.16	0.08	0
69.91667	1.136667	18.86667	0.16	0	0.08	0.08	0.08
65.10667	1.513333	19.40333	0.16	0.08	0	0.08	0.08
67.76333	0.643333	20.15667	0.16	0.08	0.08	0	0.08
65.49667	1.73	20.37333	0.16	0.08	0.08	0.08	0
63.86667	1.81	20.13	0.08	0	0.16	0.16	0
65.42	1.366667	21.55	0.08	0	0.16	0.08	0.08
64.71667	1.663333	18.87333	0.08	0	0.08	0.16	0.08
65.76667	1.56	20.55333	0.08	0	0.08	0.08	0.16

**Table 2:** Examples of actual output and expected output of experiment.

Actual output					Expected output				
*A*1	*A*4	*B*4	*C*4	*D*4	*A*1	*A*4	*B*4	*C*4	*D*4
0.1605	0.0566	0.0262	0.0412	0.0576	0.24	0.08	0.08	0	0
0.0009	0.1468	0.1755	0.0317	0.044	0	0	0	0.4	0
0.0045	0.1113	0.0952	0.024	0.0419	0.16	0.08	0	0.16	0
0.3388	0.1398	0.0169	0.051	0.0468	0.16	0.08	0	0	0.16
0.0208	0.125	0.3111	0.2054	0.1481	0.08	0	0	0.08	0.24
0.2196	0.0578	0.0212	0.0427	0.0545	0.24	0	0.08	0	0.08
0.0014	0.1297	0.1619	0.026	0.0443	0.08	0.24	0	0.08	0
0.0067	0.0875	0.058	0.0116	0.0523	0.08	0.16	0.16	0	0
0.0345	0.0798	0.0281	0.0139	0.0425	0	0	0	0.08	0.32
0.0008	0.1492	0.1702	0.0285	0.0462	0	0	0.08	0.32	0
0.0227	0.0912	0.0413	0.0231	0.0424	0.16	0.16	0	0	0.08
0.0077	0.1009	0.074	0.0213	0.0421	0	0	0.16	0	0.24
0.0022	0.1204	0.094	0.0174	0.0465	0	0.24	0	0	0.16
0.0017	0.1287	0.1474	0.0258	0.0438	0	0.32	0	0.08	0
0.1147	0.0658	0.0167	0.0162	0.0393	0.32	0	0.08	0	0
0.0028	0.1242	0.1194	0.0321	0.0417	0	0	0	0.32	0.08
0.0922	0.0716	0.0166	0.0124	0.0395	0.08	0	0.24	0	0.08

**Table 3:** The MSE of BPNN.

Serial number	1	2	3	4	5	6	7	8	9	10

MSE	0.0289	0.0417	0.0406	0.0346	0.0387	0.0416	0.035	0.0368	0.0366	0.0408

**Table 4:** The MSE of GA+BP.

Serial number	1	2	3	4	5	6	7	8	9	10

MSE	0.0335	0.0327	0.0333	0.0349	0.03	0.0324	0.0318	0.0338	0.0309	0.0316
